# An international perspective on young stroke incidence and risk factors: a scoping review

**DOI:** 10.1186/s12889-024-19134-0

**Published:** 2024-06-18

**Authors:** Dinah Amoah, Matthew Schmidt, Carey Mather, Sarah Prior, Manoja P. Herath, Marie-Louise Bird

**Affiliations:** 1 School of Health Sciences, University of Tasmania, Launceston, Australia; 2 School of Nursing, University of Tasmania, Launceston, Australia; 3 Tasmanian School of Medicine, University of Tasmania, Burnie, Australia

**Keywords:** Epidemiology, Perinatal stroke, Paediatric stroke, Risk factors, Age group standardisation, Global, Under 30 years

## Abstract

**Background:**

Stroke among younger age groups is increasing globally. While there is a focus on research conducted on people under 65 years who have had a stroke, there is a paucity of data on the incidence and risk factors of stroke among younger people (≤ 30 years). This scoping review examines evidence on incidence and risk factors for perinatal, paediatric and young adult stroke globally.

**Methods:**

The review was guided by the Joanna Briggs Institute’s scoping review methodology. A systematic search was conducted on 23rd March 2022 across Medline Ovid, Embase, PsycINFO and Cumulative Index to Nursing and Allied Health Literature (CINAHL). The eligibility criteria included all study designs providing information on the incidence and risk factors of stroke among young people (≤ 30 years) in the last ten years.

**Results:**

A total of 5750 articles were identified. After screening, 471 articles (224 cohort studies (47.6%), 164 case studies/case series (34.8%), 35 reviews (7.4%), 30 case-control (6.4%) and 18 combinations of designs (3.8%) were included. There was data from 50 different countries, 199 studies were from high-income countries, upper and middle income (*n* = 38), lower middle-income (*n* = 39), low-income (*n* = 3) countries, international study (*n* = 7) and a further 185 articles did not state the country of research. Most of the studies (63%) focused on risk factors while incidence constituted 37%. Incidence data were reported heterogeneously across studies, leading to an inability to synthesise data. The three most frequently reported risk factors for perinatal stroke were infections, cardiac conditions, and intrapartum factors. Vasculopathies, infection and cardiac conditions accounted for most reported risk factors for paediatric stroke, while chronic conditions such as diabetes mellitus, vasculopathies and cardiac conditions accounted for the most reported risk factors among young adults.

**Conclusion:**

This review has highlighted different stroke risk factors for each age cohort of people under 30 years. The low number of epidemiological studies suggests that further research of this type is needed to fully understand the incidence and risk factors in young stroke. A standardised reporting of age groupings of incidence data is imperative to enable the comparison of data from different geographical locations.

**Supplementary Information:**

The online version contains supplementary material available at 10.1186/s12889-024-19134-0.

## Background

Stroke is a high-priority public health concern as its impact extends beyond the individual domains to the family, health system, and society, with many socio-economic consequences. Worldwide, stroke is reported to be the second leading cause of death and the third leading cause of disability [1]. Even though stroke is traditionally reported as a disease of older age, the incidence among younger age groups is increasing globally [2, 3].

Stroke in age groups under 65 years of age is particularly devastating as the associated disability can have long-lasting effects during life’s most productive years [4]. In addition, there is a reported impact on social interaction [5], activities of daily living [6], and vocation [5, 7–9]. Furthermore, Hajek, Yeates [10] revealed how childhood stroke affects cognitive and intellectual ability, resulting in performing below their peers of the same age. Other evidence suggests that half of all children with stroke present with neurological impairments which affect their ability to function independently [11].

Apart from the social, cognitive and physical effects of stroke, the economic impact is well documented in studies conducted in developed countries [12, 13]. According to the study conducted by Wang and colleagues [14], the mean cost of hospitalization, which includes expenses for diagnostic tests, treatments, supplies, and accommodation charges, was $20,396 for a group of 97,374 young stroke patients (18–64 years) in the United States of America (USA). Additionally, it is estimated that almost half of the stroke-related costs, such as treatment, rehabilitation, and loss of productivity in the USA may be attributed to young people with stroke by 2050 [15]. Similarly, the Australian health system incurred an estimated cost of $662.3 million on stroke, with about $210.8 million spent among the younger age group (< 65 years) in 2018–2019 [16]. These studies depict the high financial burden that extends from the stroke survivor to the health system. Hence, the need to target interventions and specific policies geared towards reducing the individual and socio-economic burden of stroke among the younger population.

Individual studies have investigated stroke incidence and risk factors for perinatal, paediatric, and young adult age groups. These studies have heterogeneously defined stroke depending on the scope of research with definitions of young stroke with age range (18-55years) [17–19], 18–45 years [20], (24–62 years) [21], (18–64 years) [22], (< 65 years) [23] (< 50 years) [24]. Paediatric stroke also has a range of categorisations, described as an age range of 1 month – 18 years [25, 26] or < 18 years [27], and perinatal stroke between 28 weeks’ gestation and 28 days [26]. These categories of stroke descriptions cover a wide range, making it difficult to collate information regarding the specific risk factors and incidence of stroke among the younger people (≤ 30 years) within the young stroke cohorts.

These young cohorts may have different aetiologies than the older adults. Further to this, the needs of young adults less than 30 years are different from those more than 30 years as this cohort are in a stage of their life making critical life decisions that might include academic and career aspirations and family based decisions. A recent qualitative study on the unmet needs of young adults revealed a preference among stroke survivors under the age of 30 for a separate social support group, distinct from those survivors over the age of 30 [13]. Keating and colleagues [12] also reported this disparity, noting that the needs of stroke survivors under the age of 35 differ significantly from those aged 35 and above. Their research indicated that younger stroke survivors were less inclined to seek help from healthcare professionals, suggesting a unique age-related variation in post-stroke support [12]. This further emphasises the importance of age-specific considerations in the provision of post-stroke care and support.

This study aims to understand the extent of available evidence on incidence and risk factors of stroke among the younger population across different countries, encompassing perinatal, paediatric and young adults. The scoping review will efficiently identify and prioritise areas for future review that will support primary and secondary preventive interventions for stroke in the young population. For this review, three age cohorts were considered: perinatal (28 weeks’ gestation–28 days), paediatric (28 days–18 years) and young adult (18–30 years).

### Scoping review research questions

The review seeks to answer the following questions:

1. What literature is available on stroke incidence and risk factors among the younger population (≤ 30 years)?
2. What are the incidence and known risk factors of stroke internationally for perinatal, paediatric, and young adult age groups?

## Methods

This review was conducted in accordance with the Joanna Briggs Institute (JBI) methodology for scoping reviews [28] and reported according to the Preferred Reporting Items for Systematic Reviews and Meta-Analyses extension for scoping review (PRISMA-ScR) [29]. See supplementary file Addendum S1 for completed PRISMA-ScR checklist. This review was registered in Open Science Framework (https://osf.io/4mcek/).

### Search strategy

A preliminary search of MEDLINE Ovid, the Cochrane Database of Systematic Reviews, Prospero, and JBI Evidence Synthesis conducted on 12th February 2022 revealed no systematic reviews or scoping reviews on the topic or in progress. Extensive literature on the incidence and risk factors of stroke among the younger population was returned in the search, indicating the feasibility of this search topic.

An initial limited search of MEDLINE Ovid and Scopus databases was undertaken to identify articles on the topic. The text contained in the titles and abstracts of relevant articles and the index terms used to describe the articles were used to develop a full search strategy in Medline Ovid in consultation with a research librarian. The search strategy was adapted for each included database (Medline Ovid, Embase, PsycINFO, CINAHL). The reference lists of identified articles were also scanned for relevant articles. All database searches were limited from January 2012 to March 2022. The following search terms and keywords were used: Cerebrovascular Accident (CVA), stroke, brain infarction, incidence, aetiology, causes, risk factors, perinatal, paediatric, young adult. See Addendum S2 for the search strategy across all four databases.

### Eligibility criteria

#### Inclusion criteria and exclusion criteria

The PCC (Population, Concept, Context) framework recommended by JBI [30] was used to identify eligible studies for this review. Table 1 represents the inclusion and exclusion criteria.

**Table 1:** Inclusion and exclusion criteria

PCC Framework	Inclusion	Exclusion
*Participants*	• Age group ≤ 30 years • Articles with an age group greater than 30 years may be included if the study refers to separate age groups for the young group • English language • Studies conducted on humans	• Age group greater than 30 years with no segregation of characteristics for the younger age groups • Non-English articles • Animal studies
*Concept*	• Studies on young adult, childhood, and perinatal stroke • Clinical diagnosis of any type of stroke explicitly stated • Studies reporting incidence, prevalence, risk factors, etiology, causes • Epidemiological studies	• Other cardiovascular diseases • Studies on acquired brain injury, traumatic brain injury, transient ischaemic attack (TIA) • Recurrent stroke
*Context*	• All countries including high, upper-middle, low and low-middle income • All genders, races • Articles published within the last ten years	

### Source of evidence selection

Following the search, all identified citations were collated and uploaded into Endnote version 20 software and then transferred to Covidence software for de-duplication and screening. Two authors screened each title and abstracts independently, using the inclusion and exclusion criteria as a guide. Potentially relevant sources were retrieved in full, and the full-text articles of the selected citations were assessed in detail against the inclusion criteria by four independent reviewers. Where data could not be accessed, the team contacted the authors with a request for additional data. Reasons for the exclusion of sources of evidence at the full-text screening stage were recorded and reported in the scoping review. Any disagreements that arose between the reviewers at each stage of the selection process were discussed, and an additional reviewer resolved the conflicts. In instances where Covidence failed to remove duplicates automatically, reviewers removed duplicates manually during the full-text screening stage. The included studies were not assessed for quality and risk of bias because this is not the priority for a scoping review [29].

### Data extraction and analysis

Data was extracted from included studies by two independent reviewers using a custom data extraction tool in Covidence. The extraction form was first piloted by one reviewer using 10 selected articles, and revision was made through discussion. The data extracted included specific details about the participants, concept, context, study methods, and key findings relevant to the review questions. Data was extracted based on the following headings: author, year of publication, aim, study design, category of stroke by age group, type of stroke, risk factors, incidence, aetiology, country and level of income (high, upper-middle, low and low-middle income) based on the World Bank’s 2020 country classification [31] to ascertain the geographical scope of the topic. Any disagreements between reviewers were resolved through discussion and validated by a third reviewer. No meta-analysis was undertaken for this review. Extracted data was exported to Microsoft Excel (Microsoft 365 version), analysed and presented using descriptive statistics. A narrative summary is presented below to accompany the tabulated results.

### Digressions from protocol

The age group was extended from < 25 to ≤ 30 years as it was challenging to obtain data on the age group 18–25 years old. Also, the title “aetiology” of stroke was changed to “risk factors” as the literature reviewed interchanged risk factors and aetiology, and it was deemed necessary to capture both risk factors and aetiology under risk factors because ascertaining the actual cause was not well described.

## Results

**Fig. 1:**
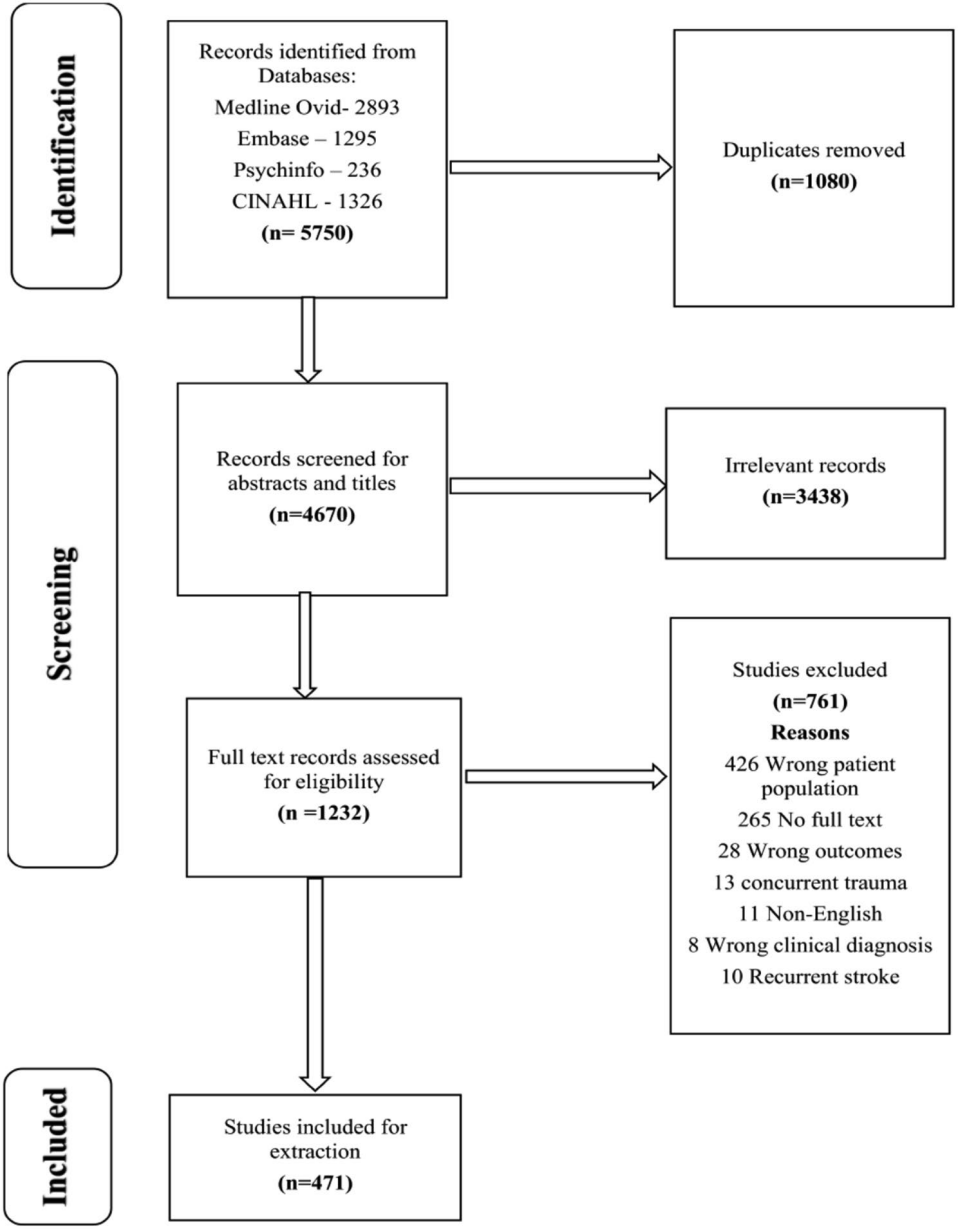
Preferred reporting items for systematic reviews and meta-analyses extension for scoping review (PRISMA-ScR) flow diagram

Search of four databases identified a total of 5750 articles. After screening and removal of duplicates, 471 articles were included in this review. Details of search results per databases have been reported (Fig. 1) in line with the Preferred Reporting Items for Systematic Reviews and Meta-analyses extension for scoping review (PRISMA-ScR). The extracted data is reported in three sections: characteristics of included studies, risk factors of stroke and incidence of stroke. Furthermore, the retrieved data is reported individually according to age group categories of stroke, namely perinatal, paediatric, and young adults. Key indicators reported for each age group category of stroke include research design, categorisation of the number of studies by type of stroke, risk factors, countries of study, and income level.

### Characteristics of included studies

**Fig. 2:**
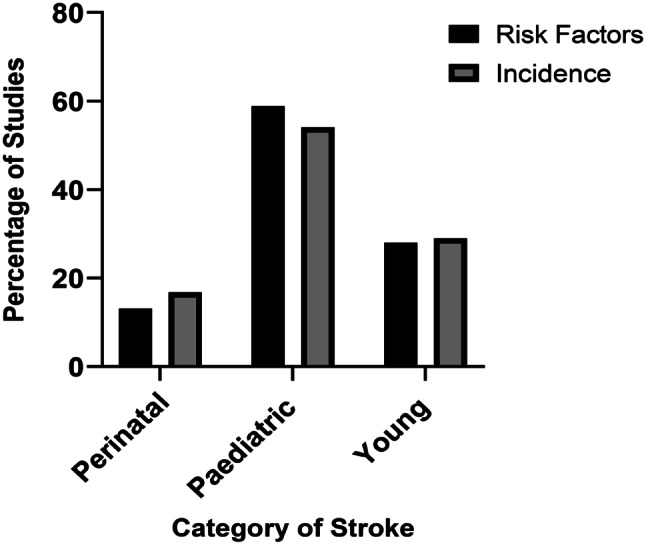
Percentage of studies on risk factors and incidence by age category

Among the 471 articles included, the year of publication ranged from 2012 to 2022, while the reported study periods were from 1969 to 2020. There were 224 (47.6%) cohort studies, 164 (34.8%) case studies/case series, 35 (7.4%) reviews, 30 (6.4%) case-control and a combination of designs constituted 18 (3.8%). Concerning total reports on incidence and risk factors, some studies reported on more than one age group, and others reported on both incidence and risk factors therefore data for studies reporting on more than one age group had to be duplicated for each age group category. The same approach was adopted for studies on both risk factors and incidence, thereby conflating the total number of studies included. In view of that, there were 822 reports on incidence and risk factors, with 518/822 (63%) reports on risk factors and 304/822 (37%) reports on incidence. A high proportion of studies (*n* = 470/822, 57.1%) were in paediatric stroke, with young stroke and perinatal stroke studies contributing (*n* = 233/822, 28.4%) and (*n* = 119/822, 14.5%) respectively. Data were retrieved from 50 different countries, with most of the studies (*n* = 199/471, 42.3%) conducted in high-income countries, studies from upper and middle income were (*n* = 38/471, 8.1%), lower middle-income (*n* = 39/471, 8.3%), low-income (*n* = 3/471, 0.6%), international study (*n* = 7/471, 1.5%) and a further 185/471 (39.3%) articles did not state the country of research. Most of the studies were conducted in the USA (*n* = 69) followed by UK (*n* = 21) and China (*n* = 19). Full details on the authors, aim, sample size, gender, and data collection method can be found in the supplementary file Addendum S3. Figure 2 shows the percentage of studies on risk factors and incidence per age group category.

### Risk factors of stroke

Risk factors are attributes, characteristics, or exposures that heighten an individual’s probability of developing a disease or health disorder [32]. Conversely, aetiology pertains to the actual cause or the specific factors that instigate the onset of a disease [33].

Despite the distinct meanings of these terms, they were often used interchangeably across studies reviewed. For example, congenital heart disease was classified as an aetiology of stroke by some authors, while others labelled it as a risk factor—this interchangeable use of terms introduced challenges in distinguishing between the two. Consequently, for this review, we included studies reporting the aetiology of stroke under the broader category of risk factors.

There were (*n* = 259/471) studies on risk factors and (*n* = 39/471) on both incidence and risk factors. The studies that reported on both the incidence and risk factors were included in the group of studies that focused on risk factors. In addition, studies reporting on more than one age group were also duplicated therefore total reports on risk factors for stroke was 518/822 (63%) across the age groups. There were varying ways studies reported the risk factors of stroke. For example, some research reported risk factors based on either haemorrhagic or ischaemic stroke, while others were based on general stroke, including both types of stroke. The reporting of the risk factors was categorised based on the age groups.

### Risk factors for perinatal stroke

Out of the total studies on risk factors, 68 out of 518 (13.1%) were reported on perinatal stroke. The majority of these studies were cohort studies (58.8%, 40/68), while case-control studies made up the smallest proportion (10.2%, 7/68). Furthermore, a considerable proportion of the studies (48.5%, 33/68) focused on ischaemic stroke. Studies from high-income countries accounted for most of the data on risk factors for perinatal stroke and the top four (4) countries reporting were the USA (*n* = 14), United Kingdom (UK) (*n* = 4), Canada (*n* = 4) and Australia (*n* = 4). There were multiple risk factors for perinatal stroke and the top three (3) frequently reported were infections (neonatal sepsis, meningitis, necrotising enterocolitis, maternal sepsis), cardiac conditions (congenital heart disease, cardiac defects, aortic coarctation) and intrapartum/foetal related factors (Apgar score < 7 at 5 min, tight nuchal cord, vacuum delivery). Table 2 provides a summary of risk factors for perinatal stroke. Further details of study characteristics can be found in supplementary file Addendum S4.

**Table 2:** Summary of risk factors for perinatal stroke

Category	Number of studies	Number of case reports
Infection/Inflammatory Disease	25	4
Cardiac Conditions	21	0
Intrapartum And Foetal Related Factors	21	0
Maternal Related Factors	17	1
Vasculopathy	15	1
Surgical Procedure	9	0
Genetic Disorder	9	1
Prothrombotic Disorders	6	0
Malignancies	5	0
Haemolytic Disorders	4	0
Unknown	4	0
Autoimmune	3	0
Metabolic And Endocrine Disorders	2	0
Medication/ Drug	1	0

### Risk factors for paediatric stroke

Risk factors for paediatric stroke were reported in 305/518 (58.9%) of total studies on risk factors. Of paediatric stroke studies reviewed, cohorts and case reports accounted for 146/305 (47.9%) and 100/305 (32.8%), respectively. There were 140/305 (45.9%) studies on ischaemic strokes, 72 on haemorrhagic strokes, and 93 on both haemorrhagic and ischaemic strokes. Most of the studies (*n* = 128/305, 41.9%) were conducted in high-income countries, with only one study from a low-income country; 124/305 (40.7%) of the paediatric stroke studies had the country of research not stated. The top four (4) countries reporting data on risk factors for paediatric stroke were the USA (*n* = 43), China (*n* = 13), the UK (*n* = 10) and Canada (*n* = 10). There were multiple risk factors for paediatric stroke and the top three (3) frequently reported were vasculopathies (arteriovenous malformation, Moyamoya disease, cervical arterial dissection), infections (otitis media, Human immunodeficiency Virus (HIV) infection, meningoencephalitis) and cardiac conditions (aortic coarctation, patent foramen ovale, cardiomyopathy). Table 3 provides a summary of risk factors for paediatric stroke. Further details of study characteristics can be found in supplementary file Addendum S5.

**Table 3:** Summary of risk factors for paediatric stroke

Category	Number of studies	Number of case reports
Vasculopathy	89	18
Infection/inflammatory disease	81	25
Cardiac conditions	46	3
Procedure (surgical, diagnostics)	42	3
Chronic conditions	41	9
Haemolytic disorders	39	5
Prothrombotic disorders	35	7
Malignancies	34	7
Undetermined	23	0
Genetic disorders	19	12
Trauma	19	2
Medication / drug	15	5
Metabolic and endocrine disorders	10	4
Autoimmune	9	1
Foetal conditions	8	1
Pregnancy and delivery-related factors	8	1
Other	3	3
Vaccination	2	0
Socio-economic factors	1	0

### Risk factors for young stroke

Risk factors for young stroke were reported in 145/518 (28.0%) of the total studies on risk factors. Out of the young stroke studies reviewed, cohorts and case reports accounted for 71/145 (48.9%) and 55/145 (37.9%), respectively. Studies conducted on ischaemic stroke constituted a high proportion of 55 (45.9%) with 42 haemorrhagic strokes and 48 (both haemorrhagic and ischaemic stroke). Studies from high-income countries accounted for majority 60/145 (41.3%) of the studies, with only one study from a low-income country. Almost a quarter (*n* = 52/145, 35.9%) of the young stroke studies did not state the country of research. The top four (4) countries reporting data on risk factors for young stroke were the USA (*n* = 14), China (*n* = 9), India (*n* = 7) and UK (*n* = 6). There were multiple risk factors for young stroke and the top three (3) frequently reported were chronic conditions (diabetes mellitus, hypertension, migraine, nephrotic syndrome), vasculopathies (arteriovenous malformation, cerebral artery aneurysm, Moyamoya disease) and cardiac conditions (coarctation of the aorta, congenital heart disease, atrial fibrillation). Table 4 provides a summary of risk factors for young stroke. Further details of study characteristics can be found in supplementary file Addendum S6.

**Table 4:** Summary of risk factors for young adult stroke

Category	Number of articles	Number of case reports
Chronic conditions	30	4
Vasculopathy	22	4
Cardiac conditions	20	2
Medication/ drug	18	12
Infection/inflammatory disorders	17	8
Metabolic and endocrine disorders	14	3
Malignancies	11	5
Haemolytic disorders	11	0
Genetic disorder	9	4
Pregnancy and delivery related factors	9	2
Prothrombotic disorders	9	0
Surgical procedure	8	0
Undetermined	8	0
Lifestyle	6	0
Socio-environmental factors	5	2
Trauma	5	2
Autoimmune disorders	4	1
Other	2	2

### Incidence of stroke

Data on the incidence of stroke among the younger population ≤ 30 years old was reported in 304/822 studies. Some studies reported incidence data only while others reported on both incidence and risk factors. Each of the studies on both incidence and risk factors were duplicated for risk factors and incidence. Hence, the high number of studies exceeding the overall total. Data on the incidence of paediatric stroke was the most reported (*n* = 165/304, 54.3% ), followed by young stroke (*n* = 88/304, 28.9%) and perinatal stroke (*n* = 51/304, 16.8%).

### Incidence by stroke type

**Fig. 3:**
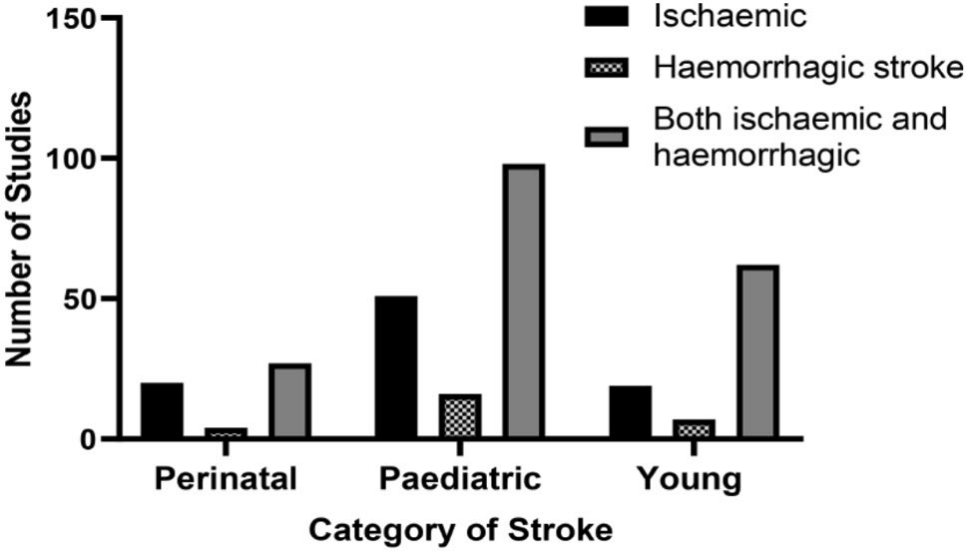
Number of studies on the incidence of stroke by stroke type and age category

Most of the incidence data reported were on both haemorrhagic and ischaemic stroke across all categories of stroke. Types of stroke by category is presented in Fig. 3.

### Incidence of stroke by income level

Majority (*n* = 187/304, 61.5%) of the incidence data were from high-income countries. The top 3 reported countries for perinatal stroke were USA (*n* = 9), Canada (*n* = 6) and Australia (*n* = 4). For paediatric stroke studies, USA (*n* = 35), Canada (*n* = 10) and China (*n* = 8) were the top 3 most reported countries. USA (*n* = 9), Australia (*n* = 6) and China (*n* = 6) were the top 3 countries reporting the incidence of stroke among young people.

### Calculation of incidence of stroke across perinatal, paediatric and young adult age groups

**Fig. 4:**
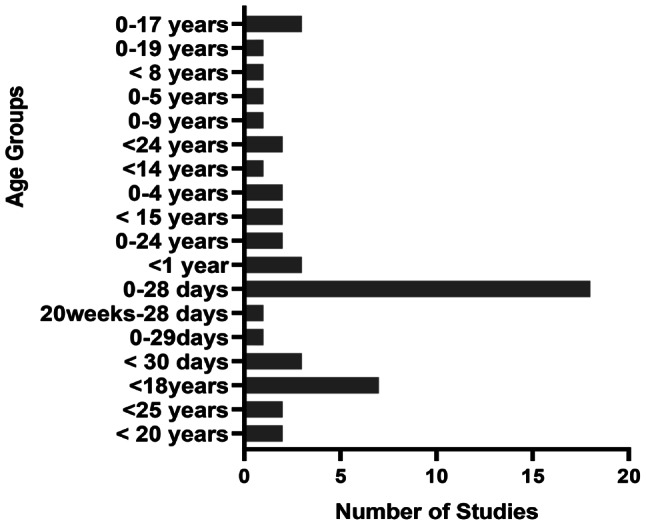
Heterogeneity of reporting perinatal stroke incidence data

**Fig. 5:**
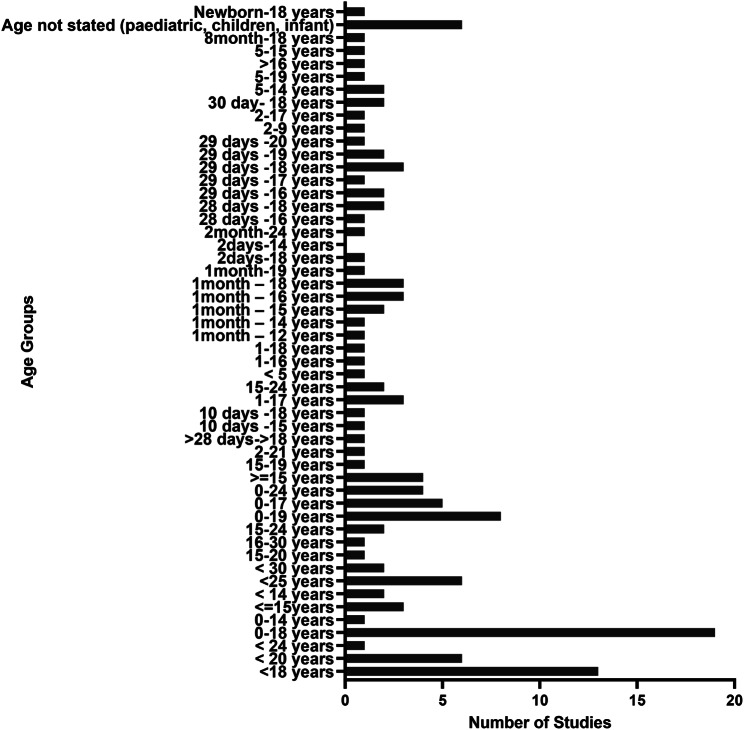
Heterogeneity of reporting paediatric stroke incidence data

**Fig. 6:**
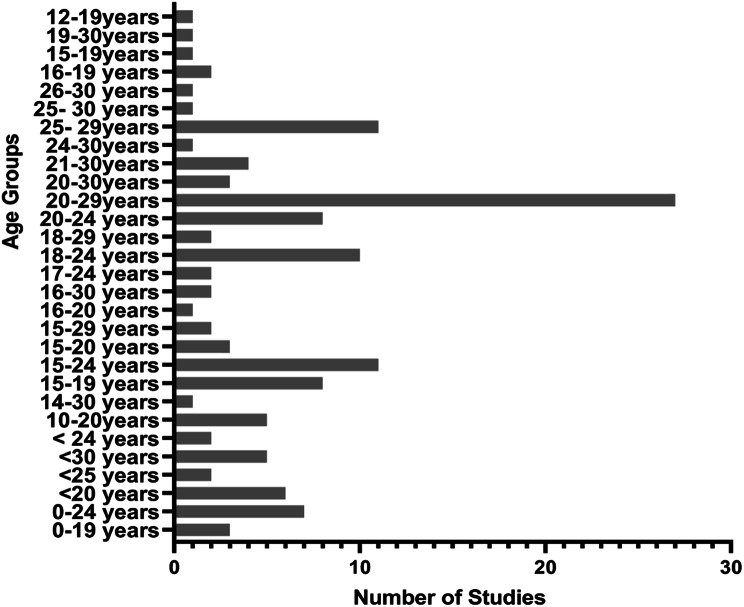
Heterogeneity of reporting young stroke incidence data

Stroke incidence data were heterogeneously reported across all categories of stroke in terms of the unit of measurement, population sampled and age groupings. Studies reporting on the incidence of stroke across perinatal, paediatric, and young adults had varying age ranges. Figures 4 and 5, and 6 present details about variations in age groupings for incidence data.

### Heterogeneity of reporting based on the unit of measurement and sampled population

**Fig. 7:**
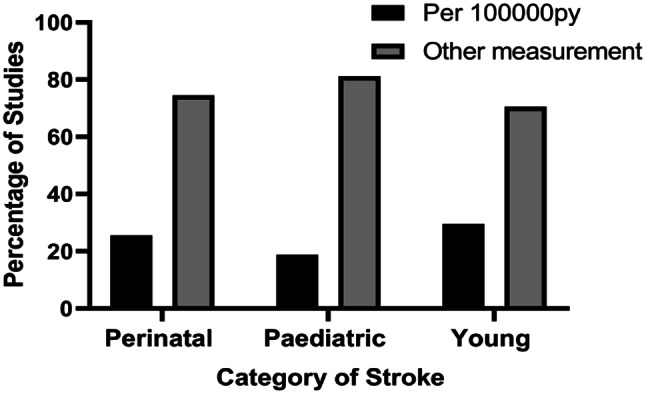
Summary of the Percentage of Studies on Incidence Per Unit of Measurement

The reporting of incidence across all age categories varied, including a mix of hospital-based [34–38] and population-based data [39–41] with measurements such as per 100,000 person-years (py), per 1000 population, percentages and absolute numbers. Table 5 provides details of the variations in the unit of measure across the categories of stroke. A summary of heterogeneity across stroke categories is presented in Fig. 7.

**Table 5:** Heterogeneity of reporting based on units of measurement

Category	No. Studies per 100,000 per year	No. Studies per 1000 Population	No. Studies in Percentage	No. Studies in Numbers
Perinatal stroke	13	6	30	2
Paediatric stroke	31	4	103	27
Young stroke	26	3	41	18

**Other measurement includes percentages, absolute numbers, per 1000 LB, per 1000 births, per million admissions.

## Discussion

This scoping review aimed to examine evidence on the incidence and risk factors for perinatal, childhood and young adult stroke globally over the last decade. The review revealed heterogeneity in reporting young stroke data and multiple risk factors for perinatal, paediatric and young adult stroke.

Although previous studies have reported that one-third of the causes of stroke among the young population are cryptogenic [42, 43], this review has highlighted multiple risk factors that could increase the risk of stroke for young people ≤ 30 years. These multiple risk factors categorised broadly based on the pathophysiological mechanisms were found to be similar across most age group categories of stroke, however, variations existed in the specific risk factors for each age group category. This finding is consistent with a recent multicentre prospective cohort study conducted in 17 hospitals in the Netherlands involving 1322 patients which revealed multiple risk factors for young stroke survivors with differences in the causes of stroke between age groups [44]. This cohort study focused on ischaemic stroke and limited its sample to individuals aged 18–49 years, whereas the present review analysed data for both ischaemic and haemorrhagic types of stroke in young people aged ≤ 30 years. Understanding differences in stroke risk factors among young people could contribute to effectively implementing primary and secondary prevention measures. This review highlights the need for healthcare professionals to widen their perspective on stroke risk factors in order to optimise treatment and decrease recurrence. Furthermore, findings suggest that public health initiatives such as screening, education, and counselling should begin in childhood and be patient-centred based on age group categories rather than a composite intervention for all young people. A further evaluation of these multiple risk factors is also warranted to identify additional causes of stroke among young adults.

This review has highlighted heterogeneity in the reporting of incidence of stroke among ≤ 30 years age group in terms of age range classification, population sampled, and units of measurement used. This finding supports literature presented in previous reviews in cohorts aged 18–50 years, which revealed heterogeneity across subgroups, population selected, and definitions [4, 45, 46]. Due to the vast heterogeneity in the reporting of the incidence data, it was not possible to meaningfully synthesise the data to have an overall incidence of stroke across perinatal, paediatric and young adults. Aggregating heterogeneous data is likely to produce misleading results, as supported by a previous study that reported how using different denominators resulted in relevant differences in incident data [35]. Also, even though hospital-based data can yield important clues about the epidemiology of a condition, its usage can potentially introduce selection bias, thereby leaving out stroke clients who do not report to the hospital. This issue confirms the Cochrane library’s recommendation that merging clinically diverse studies is misleading and generates more problems than it solves [47]. Considering the socio-economic impact of stroke in the younger population, a robust data on the incidence of stroke is imperative to aid in health care planning, equitable distribution of resources and comparison across different geographical locations. In view of this, the review, therefore, recommends a standardised age grouping and unit of measurement to report accurately and separately the data for young stroke survivors.

Furthermore, the review demonstrated that published literature on stroke incidence and risk factors among young people ≤ 30 years is disproportionately centred in high-income countries with relatively few studies reported from low and low and middle-income countries (LMIC). This finding corroborates a global survey on stroke services across countries with various income levels which reported a few surveillance activities, including the presence of stroke registries, recent risk factors surveys and participation in research among low and LMIC compared to high-income countries [48]. The limited number of studies on young stroke is of concern as previous studies have reported a high burden of stroke in these low-income and LMIC [1, 49]. The reason for the low number of studies across these countries is unclear and could be due to a lack of awareness of the problem or lack of resources, including access to publication in English language journals. Even though some inferences can be made from the high-income countries, there is the need for more research on the incidence and risk factors of stroke among this cohort in order to bridge the equity gap in low-income and low-middle income countries, as there exist differences in the proportion of specific risk factors and incidence across geographical locations [50]. In addition, about 40% of total studies retrieved did not state the country of research and this finding increases the challenge to ascertain specific risk factors and incidence related to a geographical location, thereby affecting comparison across geographical areas.

Furthermore, the review identified a high number of articles on case reports/case series that deal with individuals or events and have limited generalisability to wider populations [51]. However, the high number of case reports depicts the complexity of young stroke with its unique features and comorbidities as the aims of case reports are to report unexpected events in the course of observing a condition coupled with unique features [52]. This finding highlights the need for healthcare professionals to adopt a different approach in the management and treatment of stroke among young people due to their uniqueness. In addition, the low number of epidemiological studies suggests that further research of this type is needed to fully understand the incidence and risk factors of young stroke.

The strength of this study lies in the comprehensive nature of the search, including all study designs over the last decade thereby increasing access to majority of the studies on risk factors and incidence of stroke among young people ≤ 30 years old. This review adds to other reviews that have reported important risk factors and incidence of stroke among younger populations.

### Limitations

There are some limitations to the scoping review process. Due to the large number of citations identified (5750), unpublished literature was not searched; this omission was a digression from the protocol; however, only half of the published scoping reviews in the literature do an extensive search for grey literature [53].

Furthermore, scholarly literature searches were also limited to documents in English, which may also reduce the scope of this study as language of publication inhibits geographical range of studies reviewed.

## Conclusion

This scoping review explored stroke incidence and risk factors among the younger (≤ 30 years) population across a large number of countries. The research has highlighted different risk factors for age cohorts of people under 30 years who have had a stroke. The low number of epidemiological studies suggests that further research of this type is needed to fully understand the incidence and risk factors of young stroke, especially in low and low-middle income countries. Finally, a standardised reporting of age groupings and unit of measurement is recommended to report accurately and separately the data for young stroke survivors. This will facilitate comparison of data from different geographical locations.

### Electronic supplementary material

Below is the link to the electronic supplementary material.

Supplementary Material 1

Supplementary Material 2

Supplementary Material 3

Supplementary Material 4

Supplementary Material 5

Supplementary Material 6

## Data Availability

The datasets used and/or analysed during the current study are included in this published article and its supplementary information files (Additional files S1– S6).
