# Supplementary material for: An international perspective on young stroke incidence and risk factors: a scoping review
Source: BMC Public Health. 2024 Jun 18;24:1627. doi: 10.1186/s12889-024-19134-0 (PMC11186079; doi:10.1186/s12889-024-19134-0)
Supplement: Supplementary file 2 — Supplementary Material 2 [file 12889_2024_19134_MOESM2_ESM.docx]

**Addendum 2 Search Strategies across databases**

**APA PsycINFO <1806 to March Week 3 2022>**

| **NO.** | **SEARCH STRATEGY** | **RESULT** |
| --- | --- | --- |
| 1 | (Prevalence or "demographic characteristics" or incidence or "risk factors" or etiology or aetiology or cause or epidemiology).mp. [mp=title, abstract, heading word, table of contents, key concepts, original title, tests & measures, mesh word] | 519003 |
| 2 | ("Brain Attack" or "Cerebrovascular Accident" or CVA or "Ischaemic stroke" or "Brain infarction" or Apoplexy or Thromboemboli or "Brain embolism" or "Post-stroke" or Intracerebral or "Cerebral Venous sinus Thrombosis" or "Arterial ischaemic stroke" or "Haemorrhagic stroke" or "Cerebral infarction").mp. [mp=title, abstract, heading word, table of contents, key concepts, original title, tests & measures, mesh word] | 13508 |
| 3 | (Young or Child* or Fetal or Perinatal or Neonatal or Paediatric or Adolescent or "Young Adult" or Infant or Teenage*).mp. [mp=title, abstract, heading word, table of contents, key concepts, original title, tests & measures, mesh word] | 1311664 |
| 4 | 1 and 2 and 3 | 483 |
| 5 | limit 4 to humans | 483 |
| 6 | limit 5 to English language | 463 |
| 7 | limit 6 to last 10 years | **236** |

**Cumulative Index to Nursing and Allied Health Literature (CINAHL) Search History -24-03-2022**

| **#** | **Query** | **Limiters/Expanders** | **Last Run Via** | **Results** |
| --- | --- | --- | --- | --- |
| S5 | S1 AND S2 AND S3 | **Limiters** - Published Date: 20120101-20221231; English Language; Human  **Expanders** - Apply equivalent subjects  **Search modes** - Boolean/Phrase | Interface-EBSCOhost Research Databases Search Screen - Advanced Search Database - CINAHL Complete | 1,326 |
| S4 | S1 AND S2 AND S3 | **Expander**s - Apply equivalent subjects **Search modes** - Boolean/Phrase | Interface -EBSCOhost Research Databases Search Screen - Advanced Search Database - CINAHL Complete | 3,268 |
| S3 | Prevalence OR "demographic characteristics" OR incidence OR "risk factors" OR etiology OR aetiology OR cause OR epidemiology | **Expanders** - Apply equivalent subjects **Search modes** - Boolean/Phrase | Interface -EBSCOhost Research Databases Search Screen - Advanced Search Database - CINAHL Complete | 1,585,308 |
| S2 | "young people" OR youth OR adolescents OR young adults OR teenagers OR children OR perinatal OR paediatric OR pediatric OR neonat* | **Expanders** - Apply equivalent subjects **Search modes** - Boolean/Phrase | Interface - EBSCOhost Research Databases Search Screen - Advanced Search Database - CINAHL Complete | 1,265,614 |
| S1 | "Brain Attack" OR "Cerebrovascular Accident" OR CVA OR "Ischaemic stroke" OR "Brain infarction" OR Apoplexy OR Thromboemboli OR "Brain embolism" OR "Post-stroke" OR Intracerebral OR "Cerebral Venous sinus Thrombosis" OR "Arterial ischaemic stroke" OR "Haemorrhagic stroke" OR "Cerebral infarction" | **Expanders** - Apply equivalent subjects **Search modes** - Boolean/Phrase | Interface -EBSCOhost Research Databases Search Screen - Advanced Search Database - CINAHL Complete | 78,963 |

**Embase <1974 to 2022 March 23>**

| **NO.** | **SEARCH STRATEGY** | **RESULT** |
| --- | --- | --- |
| 1 | ("Brain Attack" or "Cerebrovascular Accident" or CVA or "Ischaemic stroke" or "Brain infarction" or Apoplexy or Thromboemboli or "Brain embolism" or "Post-stroke" or Intracerebral or "Cerebral Venous sinus Thrombosis" or "Arterial ischaemic stroke" or "Haemorrhagic stroke" or "Cerebral infarction").ab,kw,ti. | 127593 |
| 2 | (Prevalence or "demographic characteristics" or incidence or "risk factors" or etiology or aetiology or cause or epidemiology).ab,kw,ti. | 4353602 |
| 3 | ("young people" or youth or adolescents or "young adults" or teenagers or children or perinatal or paediatric or pediatric or neonat*). ab,kw,ti | 2379432 |
| 4 | 1 and 2 and 3 | 2460 |
| 5 | limit 4 to (human and English language and last 10 years) | **1295** |

**Ovid MEDLINE(R) ALL <1946 to March 22, 2022>**

| **NO.** | **SEARCH STRATEGY** | **RESULT** |
| --- | --- | --- |
| 1 | (Prevalence or "demographic characteristics" or incidence or "risk factors" or etiology or aetiology or cause or epidemiology).mp. [mp=title, abstract, original title, name of substance word, subject heading word, floating sub-heading word, keyword heading word, organism supplementary concept word, protocol supplementary concept word, rare disease supplementary concept word, unique identifier, synonyms] | 6392768 |
| 2 | ("Brain Attack" or "Cerebrovascular Accident" or CVA or "Ischaemic stroke" or "Brain infarction" or Apoplexy or Thromboemboli or "Brain embolism" or "Post-stroke" or Intracerebral or "Cerebral Venous sinus Thrombosis" or "Arterial ischaemic stroke" or "Haemorrhagic stroke" or "Cerebral infarction").mp. [mp=title, abstract, original title, name of substance word, subject heading word, floating sub-heading word, keyword heading word, organism supplementary concept word, protocol supplementary concept word, rare disease supplementary concept word, unique identifier, synonyms] | 103013 |
| 3 | (Young or Child* or Fetal or Perinatal or Neonatal or Paediatric or Adolescent or "Young Adult" or Infant or Teenage*).mp. [mp=title, abstract, original title, name of substance word, subject heading word, floating sub-heading word, keyword heading word, organism supplementary concept word, protocol supplementary concept word, rare disease supplementary concept word, unique identifier, synonyms] | 5290534 |
| 4 | 1 and 2 and 3 | 8691 |
| 5 | limit 4 to humans | 8139 |
| 6 | limit 5 to English language | 6953 |
| 7 | limit 6 to last 10 years | **2893** |
